# The Difference between Anxiolytic and Anxiogenic Effects Induced by Acute and Chronic Alcohol Exposure and Changes in Associative Learning and Memory Based on Color Preference and the Cause of Parkinson-Like Behaviors in Zebrafish

**DOI:** 10.1371/journal.pone.0141134

**Published:** 2015-11-11

**Authors:** Xiang Li, Xu Li, Yi-Xiang Li, Yuan Zhang, Di Chen, Ming-Zhu Sun, Xin Zhao, Dong-Yan Chen, Xi-Zeng Feng

**Affiliations:** 1 State Key Laboratory of Medicinal Chemical Biology, Key Laboratory of Bioactive Materials, Ministry of Education, College of Life Science, Nankai University, Tianjin 300071, China; 2 The Institute of Robotics and Automatic Information Systems, Nankai University, Tianjin 300071, China; 3 The Key Laboratory of Animal Models and Degenerative Diseases, Department of Physiology, School of Medicine, Nankai University, Tianjin, 300071, China; Karlsruhe Institute of Technology, GERMANY

## Abstract

We describe an interdisciplinary comparison of the effects of acute and chronic alcohol exposure in terms of their disturbance of light, dark and color preferences and the occurrence of Parkinson-like behavior in zebrafish through computer visual tracking, data mining, and behavioral and physiological analyses. We found that zebrafish in anxiolytic and anxious states, which are induced by acute and chronic repeated alcohol exposure, respectively, display distinct emotional reactions in light/dark preference tests as well as distinct learning and memory abilities in color-enhanced conditional place preference (CPP) tests. Additionally, compared with the chronic alcohol (1.0%) treatment, acute alcohol exposure had a significant, dose-dependent effect on anxiety, learning and memory (color preference) as well as locomotive activities. Acute exposure doses (0.5%, 1.0%, and 1.5%) generated an “inverted V” dose-dependent pattern in all of the behavioral parameters, with 1.0% having the greatest effect, while the chronic treatment had a moderate effect. Furthermore, by measuring locomotive activity, learning and memory performance, the number of dopaminergic neurons, tyrosine hydroxylase expression, and the change in the photoreceptors in the retina, we found that acute and chronic alcohol exposure induced varying degrees of Parkinson-like symptoms in zebrafish. Taken together, these results illuminated the behavioral and physiological mechanisms underlying the changes associated with learning and memory and the cause of potential Parkinson-like behaviors in zebrafish due to acute and chronic alcohol exposure.

## Introduction

The abuse or misuse of alcohol, such as alcoholism or alcohol dependence syndrome, has many negative effects on society and individual people [1]. According to annual statistics, alcoholism results in thousands of deaths and financial losses of more than $150 billion in the USA alone [2]. Worse still, acute excessive consumption or chronic alcohol abuse can interact with multiple biochemical targets in several regions of the brain and affect mood, learning and memory [3]. The acute consumption of alcohol at relatively low concentrations may cause some “pleasurable” effects, such as euphoria, relaxation, and the relief of stress or anxiety [4] while chronic alcohol intake results in dependence, which manifests as increased stress and anxiety without alcohol [5]. Therefore, alcoholism not only negatively affects addictive behaviors but anxious (stress and anxiety) and advanced cognitive behaviors (learning and memory) as well [6]. However, the behavioral, psychological and physiological mechanisms underlying the effect of alcohol on human neural physiological processes has remained elusive [7].

To elucidate the underlying mechanism through which alcohol affects cognitive behaviors, multiple animal models have been employed, among which rodent models are the most common [7], but inferior model organisms, such as the fruit fly (*Drosophila melanogaster*)[8, 9] and *Caenorhabditis elegans* [10], have aroused the interest of researchers as well. The zebrafish (*Danio rerio*) has recently been proposed as an appropriate tool for the behavioral, biological and genetic analysis of the effects of alcohol [11]. Generally, the zebrafish possesses some unique advantages over other laboratory organisms due to its balance between system complexity (it is a vertebrate) and practical simplicity (it is easy to breed and keep in large numbers at low cost)[12]. Specifically, the use of zebrafish appears to be prominent in the study of neural behavior. First, zebrafish gene sequences have indicated genetic homology of nearly 70% or higher than that of mammals, including humans [13], and the research of Chatterjee and Gerlai demonstrated that the neurotransmitter systems of zebrafish and mammals are similar [14]. In particular, due to the unique homology in the neurotransmitter receptors compared to mammals, zebrafish are able to be used in pharmacological screenings of drugs designed for humans [15]. Furthermore, although its brain is substantially more primitive than that of mammals, the basic neuroanatomical layout of the zebrafish is typical of vertebrates [16]. Finally, the species has a sophisticated behavioral repertoire that enables precise quantification of functional [17] behavioral changes by whatever experimental method (e.g., genetic, pharmacological, and environmental manipulations)[18, 19]. Therefore, there is great potential for the zebrafish to be a promising animal model for the study of alcohol -induced behavioral changes as well as an efficient behavioral and physiological tool for discovering the molecular and neural underpinnings of the negative impacts of alcohol on the neuro-system [17, 20–23]. From the behavioral, psychological and physiological perspectives, a large number of researchers have conducted many experiments to decipher the mechanisms through which alcohol affects zebrafish. Numerous behavioral changes have been reported, including the alteration of social behaviors [17, 19, 24–26], fear responses [23], anxiety responses [27], neurochemical levels [27] and gene expression. Furthermore, experimental methods and devices, such as light/dark boxes [6], conditioned place preference (CPP) [28, 29], and T or Y mazes [30] have been employed. Despite the rationale for the use of zebrafish in the genetic analysis of behavior and brain function in general and alcohol research in particular, the understanding and characterization of zebrafish behavior is in its infancy. There is still a paucity of data for some neuro-behaviors and ambiguity about some of the effects of alcohol on zebrafish.

Interest in the use of zebrafish in behavioral neuroscience has been increasing due to its value in learning and memory research, such as of associative and non-associative learning, which has been demonstrated by many researchers [31–35]. The color-enhanced CPP test is a new type of learning and memory test based on the natural bias of zebrafish towards specific colors, which may lead to changes in visual discrimination learning, memory and decision making [36]. Although many previous studies have assessed the neuro-effects of alcohol on diverse behavioral functions in zebrafish, relatively few studies have evaluated the impacts of alcohol on advanced neuro behaviors, specifically learning and memory. For example, Priya Mathur [37] found that acute alcohol exposure relieved anxiety while chronic exposure increased anxiety in light/dark choice tests, but the author did not evaluate the different effects of acute and chronic alcohol exposure on color-associative learning and memory in zebrafish in the two distinct emotional states related to anxiety. Furthermore, Diana M. Chacon used a traditional conditioning test to evaluate the effect of alcohol on zebrafish performance by associating an unconditioned stimulus (food) to a conditioned stimulus (light)[38]. In this study, we used the color-enhanced CPP to test the effect of acute and chronic alcohol treatment on color preference in zebrafish. We were the first to use the color-enhanced CPP apparatus to discover the different effects of acute and chronic alcohol treatment on color-associative learning and memory behaviors.

It has been reported that most zebrafish learning and memory assays based on color discrimination are correlated with a visual stimulus [39]. However, there is a paucity of research examining the effects of acute and chronic alcohol treatment on color preference in zebrafish in euphoric and anxious emotional states. In this study, we relieved the anxiety of zebrafish with acute alcohol exposure (0.5%, 1.0% and 1.5%) and increased anxiety through chronic, repeated administration of alcohol (1.0%) for 8 days. A light and dark preference test was employed to verify the anxiolytic and anxiogenic effects induced by acute and chronic alcohol treatment, respectively. Second, we compared the distinct effects of acute and chronic alcohol treatment on color vision-based learning and memory behaviors of zebrafish in euphoric and anxious states via color-enhanced conditional place preference (CPP) tests by computer visual tracking. Furthermore, a pathological analysis was performed on the alcohol-targeted visual and nerve tissues, cells and proteins to detect the biological mechanisms underlying the different neuro-effects of acute and chronic alcohol exposure.

## Materials and Methods

### Experimental Animals

All of the experimental protocols and procedures involving zebrafish were approved by the Committee for Animal Experimentation of the College of Life Science at Nankai University (no. 2008) and were performed in accordance with the NIH Guide for the Care and Use of Laboratory Animals (no. 8023, revised in 1996). The zebrafish employed for each experiment (control and alcohol-treated for light/dark preference and color-enhanced conditional place preference) were obtained from the same cross and were therefore siblings. Experimental animals were maintained in aquaria at 28.5°C with a 10/14 h dark/light cycle, and 10–12 zebrafish of mixed sex were housed in each 3 L aquarium under standard conditions as previously described [40]. Only zebrafish with complete body forms, distinct stripes and scales were chosen for the behavioral and physiological experiments.

### Experimental Design and Alcohol Treatment Procedure

Zebrafish in each alcohol treatment group (0.0%, acute 0.5%, acute 1.0%, acute 1.5% and chronic 1.0%) were exposed to alcohol in groups of 12 fish and tested simultaneously in individual tanks for both the light/dark choice and color-enhanced conditioned place preference tests. The general experimental procedures are shown in **Fig E** in S1 File.

#### Acute alcohol exposure

Eight animals (for each condition) were netted gently from their 3-L housing tank and placed into a 3 L exposure tank containing 0, 0.5, 1.0 or 1.5% alcohol. The effects of acute exposure to 0.5% and 1.0% alcohol for 1 hr have been exhaustively studied in zebrafish [20, 41], and acute exposure to higher concentrations (up to 3.0%) for 20 min has been demonstrated to induce a range of relevant behavioral responses [42, 43]. Therefore, to mimic general alcohol consumption in humans, we confined the exposure time to approximately 20 min (15 min in the exposure tank + 2 min of adaptation + 5 min of recording in the individual test tank), which resulted in a brain alcohol level of ~0.14 ± 0.01 (v/v), a concentration similar to that experienced by human drinkers [17, 37]. For the acute alcohol exposure treatment, three replicates (n = 12 for one replicate) were performed for each concentration group.

#### Chronic alcohol exposure

Twelve animals each were used for the control (exposed to system water) and experimental (exposed to 1.0% alcohol) groups to assess the effect of chronic alcohol exposure. Instead of continuous exposure to alcohol, we adopted a regimen of intermittent alcohol exposure as it more closely resembles what would be experienced by human drinkers [44]. Both the control and experimental groups were moved to the behavior room at the same time each day for 8 days, and they were gently netted into exposure tanks, which were kept inside brown boxes that opened from the top, and exposed to either system water or 1.0% alcohol for 20 min. The alcohol was mixed into the water just before the fish were introduced. Following exposure, the fish were gently netted back into their housing tanks and placed back in the housing system. Three replicates (n = 12 for one replicate) were performed.

### Behavioral Tests

#### Light/Dark Choice Test

In the light/dark choice assay, an increase in the amount of time spent on the dark side is believed to reflect increased anxiety [45]. In the acute alcohol exposure experiment, each zebrafish was put into an individual light/dark choice tank (23.5×13.5×13 cm, L×W×D) with 1 L of water that contained the same alcohol concentration as the exposure tank. After a 2 min adaptation period, a 5-minute recording was made through cameras positioned above the tank, and at the end of the 5-min recording period, the zebrafish were returned to the housing tank. In the chronic exposure experiment, the light/dark choice tank only contained system water.

#### Characterization of the color papers used in color-enhanced conditional place preference test

The four colors of the papers used in this experiment were characterized with a Shimadzu UV-3600 UV-VIS-NIR photo-spectrometer (Shimadzu Co., Japan), and the reflectance spectra are shown in **Fig F** in S1 File. The peak wavelengths of the colored papers were 449.45 nm (blue), 535.37 nm (green), 577.33 nm (yellow) and 659.75 nm (red); these wavelengths are comparable to the color vision in zebrafish with peaks of ultraviolet (362 nm), violet (415 nm), cyan (480 nm) and yellow (570 nm) [46–48]. Zebrafish tested in color-enhanced CPP tests can differentiate these colors and be aware of six color combinations.

#### Color-enriched conditional place preference test

The color preference behavior and locomotive activities of the zebrafish with acute or chronic alcohol treatment were observed in the CPP apparatus [28], which consisted of a two-chambered PP box (23 ×15 ×15 cm) containing 12 cm of water (measured from the bottom). To test the color preference behavior of the zebrafish, the CPP tank was slightly modified; the black dots on one side of the bottom were replaced with different colored paper.

The behavioral experimental procedures are illustrated in **Fig E** in S1 File, and details of the pre-test training procedure for the zebrafish were provided in a previous work [28]. Each group consisted of 12 adult zebrafish that were maintained and treated in a 3-L tank for 7 days, and acute (0.5%, 1.0%, and 1.5%) and chronic (1.0%) alcohol treatments were applied. The adult zebrafish were divided into the following five treatment groups: tank water (as a control), acute 0.5%, acute 1.0%, acute 1.5% and chronic 1.0%. Beginning on the second day of the 7-day period, all of the fish were initially trained in the CPP apparatus in accordance with their group. To ensure that they became aware of the existence of the two different compartments in the CPP apparatus, the fish were habituated to the CPP with a transparent perforated barrier in the middle of the apparatus that impeded movement between the compartments.

For each dosed group, 12 fish were initially trained in the CPP, and the number of fish was halved after the shoal had explored all the compartments; the fish were given 4 min to explore the CPP during each session. Finally, we ensured that each individual fish had explored all the compartments.

On the eighth day, when all of the fish had been dosed and adapted to the CPP apparatus for 7 days, a color preference test was performed. We tested six different combinations of four colors (red and yellow, red and green, red and blue, yellow and green, yellow and blue, and green and blue). Paper of one color was attached to the bottom of one chamber, and paper of a different color was attached to the bottom of the other chamber. Each fish was tested individually during the 3 min, and its movement was tracked by a video camera for computer analysis. We evaluated the behavioral parameters (including the swimming path) of the zebrafish via self-designed zebrafish tracking software; the accuracy and availability of the software is described in the supporting material (**Figs A–D** in S1 File). Nine behavioral endpoints were statistically analyzed to determine the effects of alcohol on the cognitive and locomotive behaviors of the dosed zebrafish.

### Immunohistochemistry

After the adult zebrafish had been exposed to the alcohol, their eyes and brain tissues were harvested and immediately fixed in 4% paraformaldehyde, equilibrated in 30% sucrose/PBS overnight and embedded in OCT. Twelve-mm-thick sections were mounted on gelatin-coated slides and air dried at 37°C for at least 2 h. The tissue sections were rehydrated with PBS, blocked with 20% NGS and 2% BSA in 0.3% PBS/Triton X-1 00 (PBST) for 1 hr, and incubated with primary antibodies overnight at 4°C. The following primary antibodies and concentrations were used: mouse monoclonal antibody Zpr-1 (1:200, University of Oregon) for labelling cones and mouse monoclonal anti-tyrosine hydroxylase (1:400, Millipore, Billerica, MA) for labelling DA cells. The interpretation of the neuroanatomy follows the adult zebrafish brain atlas [49] Immunoreactions were detected using Cy3-labelled goat anti-mouse IgG diluted to 1:400 (Millipore), and the sections were counterstained with a 1:1000 dilution of 4’, 6-diamidino-2-phenylindole (DAPI) (Sigma) to label the nuclei. The slides were examined with an Olympus BX51 light microscope (Olympus, Tokyo, Japan). The images were obtained by an Olympus CCD DP71 (Olympus) and processed using Adobe Photoshop CS (Adobe Systems, San Jose, CA).

### Histopathological examination

After the adult zebrafish had been exposed to the alcohol, their eyes and brain tissues were harvested and immediately fixed in 4% paraformaldehyde. The tissues were routinely processed for paraffin embedding, and 8-mm-thick sections were cut and mounted onto glass slides. The tissue samples were stained with hematoxylin and eosin, and the sections were evaluated and photographed using an Olympus BX51 light microscope equipped with an Olympus CCD DP71 (Olympus).

### Western blot analysis

The level of tyrosine hydroxylase protein in the zebrafish brain was measured by western blot analysis. Following alcohol exposure, the brains of adult zebrafish were harvested and immediately lysed in a tissue protein extraction reagent (CWBIO, Beijing, China) and 5 mL PMSF (Sigma-Aldrich). The protein concentrations were quantified using the BCA Protein Assay Kit (CWBIO), and the proteins were subjected to SDS-PAGE and transferred onto a nitrocellulose membrane blocked with 5% non-fat dry milk in Tris-buffered saline with 0.05% Tween-20. The membrane was incubated with the following primary antibodies: mouse anti-TH (15 1000; Millipore) and mouse anti-actin (15 5000; Abmart, Shanghai, China). After being washed with Tris-buffered saline containing 0.05% Tween-20, the membrane was incubated with an anti-mouse peroxidase-conjugated secondary antibody (1:3000; CWBIO). The membrane was then washed with Tris-buffered saline containing 0.05% Tween-20, and the Super Signal West Pico chemiluminescent substrate (Thermo Scientific) was used for detection.

### Statistical analysis

To compare the same behavioral endpoint indifferently colored compartments in the CPP test, a two-sample heteroscedasticity hypothesis test was performed using Excel 2013. The level of significance was set at p<0.05 for all of the experiments, and the results are presented as the means and standard errors of the mean (SEM).

## Results

### Effects of acute and chronic alcohol treatment on the anxiety state in zebrafish

**Fig 1:**
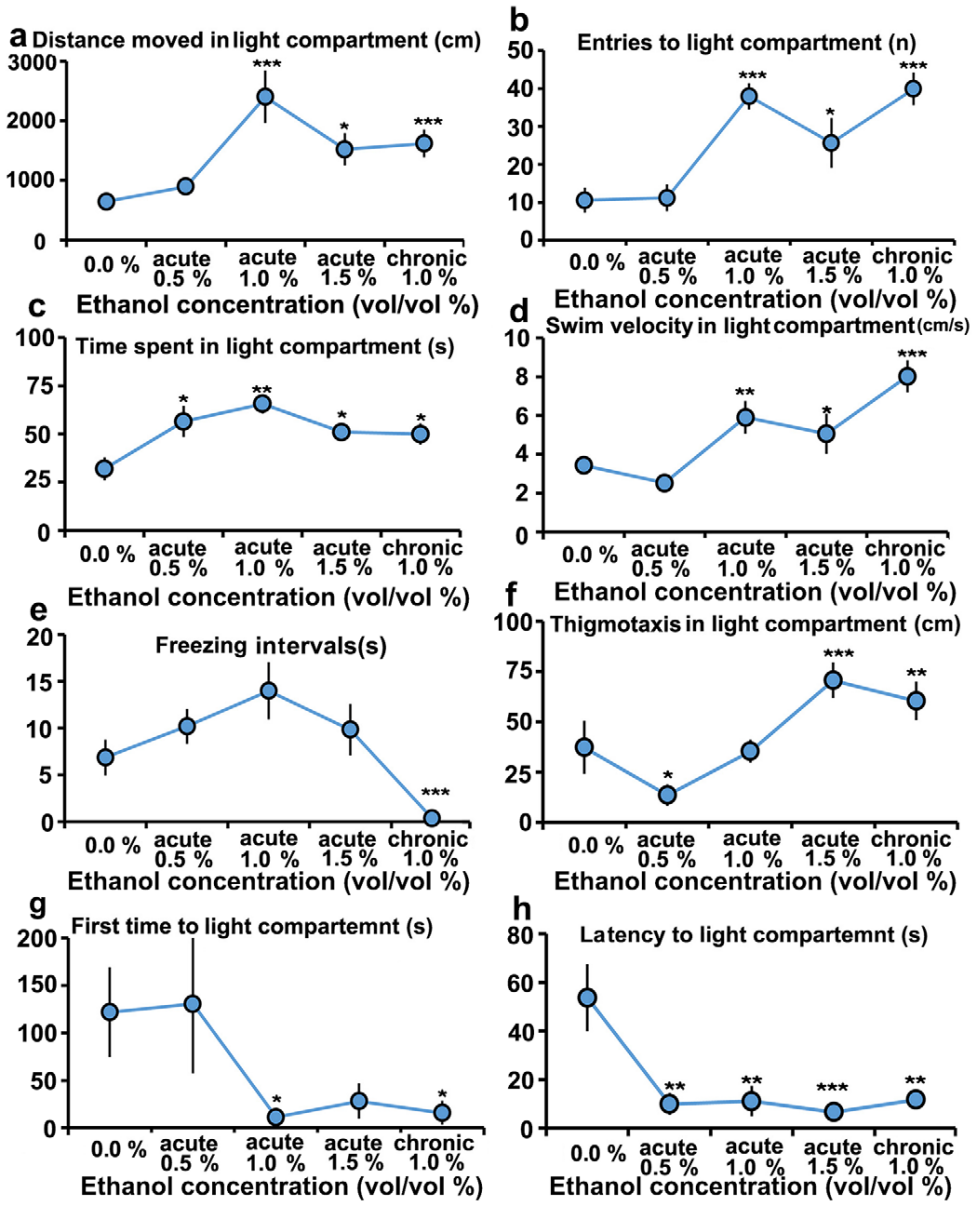
Effects of acute (0.5%, 1.0% and 1.5%) and chronic (1.0%) alcohol exposure on zebrafish behavior in light and dark preference tests. **(a)** Distance moved (cm) in the light compartment. **(b)** Entries (number of times) to the light compartment. **(c)** Time spent (s) in the light compartment. **(d)** Swim velocity (cm/s) in the light compartment. **(e)** Freezing intervals (s) in the light compartment. **(f)** Thigmotaxis (cm) in the light compartment. **(g)** First time (s) to the light compartment. **(h)** Latency (s) to the light compartment. The asterisks represent significant differences between the alcohol-treated and wild-type (0.0%) fish; an asterisk close to the balls indicates a significant difference between the treated (or untreated) and wild-type zebrafish. The data represent the means ± SEM of n = 12 zebrafish. *p < 0.05, **p <0.01, ***p <0.001.

The effects of acute and chronic alcohol exposure on eight behavioral parameters of the light/dark preference test are illustrated as ball plot graphs with each ball representing a particular acute or chronic alcohol concentration (Fig 1). Time spent or distance moved in the light or dark environment (increased time spent or distance on the dark side is believed to reflect increased anxiety [45]) has been previously applied as an indicator of anxiety-like behavior [45]. The analysis of the “Time spent in light compartment” (Fig 1a) and “Distance moved in light compartment” (Fig 1c) demonstrated that zebrafish treated with either acute 0.5%,1.0%,1.5% or chronic 1.0% alcohol spent more time in the light compartment compared to the control, indicating a general anxiolytic effect of both acute and chronic alcohol. However, compared with acute alcohol, the chronic alcohol 1.0% treatment decreased the time spent and distance moved in the light compartment, indicating a relatively anxiogenic effect of chronic alcohol. The analysis of “Freezing intervals” (Fig 1e) showed that chronic alcohol significantly increased the frequency of anxious behavior by reducing the intervals of time (s) in a freezing state. The analysis of “First time to light compartment” (Fig 1g), “Latency to light compartment” (Fig 1h) and “Entries to light compartment” (Fig 1b) indicated that both the acute and chronic alcohol treatments altered the exploratory behaviors of the zebrafish in the light compartment. In addition, the analysis of locomotor activity parameters, such as “Swim velocity in light compartment” and “Thigmotaxis in light compartment,” suggested the locomotion-promoting effect of both acute and chronic alcohol in the light compartment. In sum, these result demonstrated that 1) most of the parameters demonstrated an “inverted V” trend with acute 1.0% as a turning point and a convergent trend of acute 1.5% and chronic 1.0%. 2) Compared to the acute treatment, chronic alcohol exposure has an anxiogenic effect.

### Distinctions in learning and memory between relatively euphoric and anxious emotional states caused by acute and chronic alcohol treatments

**Fig 2:**
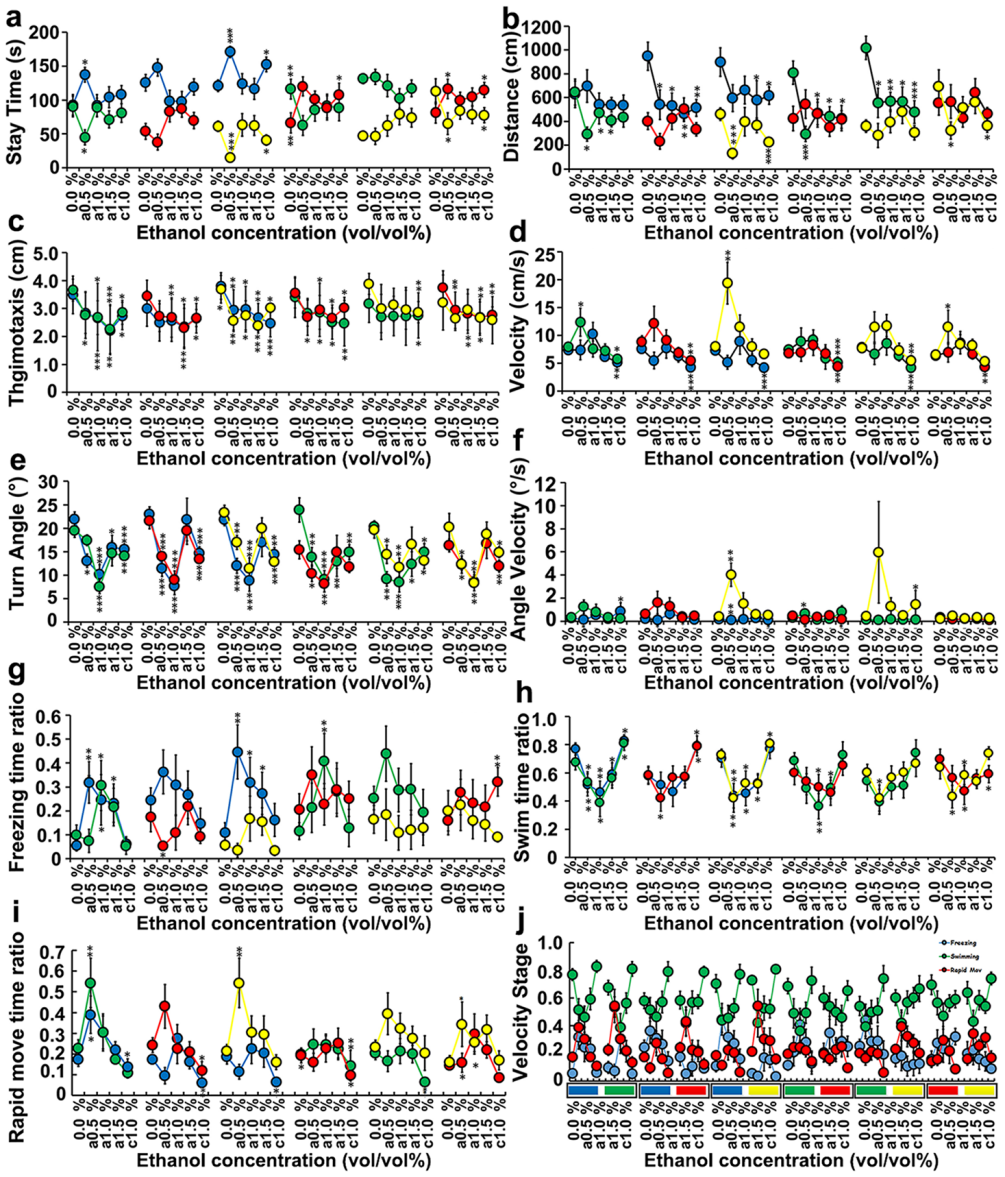
The effects of acute (0.5%, 1.0% and 1.5%) and chronic (1.0%) alcohol exposure on zebrafish behavioral parameters in six color combinations (12 color lumps) in color-enhanced conditional place preference tests. In each line chart, the six different color combinations (12 color lumps) located under the x-axis represent the different colors beneath the CPP compartments in the experiment. The five scales located under the y-axis indicate the five alcohol concentrations (vol/vol %) at which the zebrafish were treated. As shown in the line chart for each concentration, the coupled solid-colored balls represent the values of the behavioral parameters in the corresponding color compartment. The asterisks represent significant differences between the alcohol-treated and wild-type zebrafish. The data represent the means ± SEM of n = 12 zebrafish. * p < 0.05, ** p <0.01, ***p <0.001.

To identify the effects of acute and chronic alcohol treatments on all of the specific behavioral parameter in the color-enhanced CPP, we performed statistical analyses on ten behavioral parameters in six color combinations (Fig 2). In each line chart, six different color combinations (12 color lumps) are located under the x-axis, representing the different colors beneath the CPP compartments in the experiment. The five scales located under the y-axis indicate the five alcohol concentrations (vol/vol %) at which the zebrafish were treated. For every concentration, the coupled solid-colored balls represent the values of the behavioral parameters in the corresponding color compartment.

We monitored three behavioral parameters (‘‘Stay time,” ‘‘Distance” and “Thigmotaxis”) that are directly linked to color preference; an increase in the parameter value in a certain color compartment indicates the preference. It has been reported that wild-type zebrafish prefer blue and green over yellow and red [50]. For the parameter “Stay time” (Fig 2a), there was a trend in all six color combinations indicating that both the acute and chronic alcohol treatments increased zebrafish preference towards red as more time was spent in the red compartment and less time in the green, yellow and blue compartments compared with the wild-type zebrafish (control group). Still, there is a distinction between acute and chronic exposure to alcohol in the same concentration, such as 1.0%, that affects color preference. Time spent in the acute alcohol treatment (from 0.5% to 1.5%) in every color combination has the tendency to at first increase (decrease) and then decrease (increase), with acute 0.5% being the extreme, which generated an ‘inverted V’ dose-dependent pattern. Meanwhile, time spent in the chronic alcohol treatment groups in every color combination decreases (compared with the acute alcohol treatment) and increases (compared with the control). In addition, for the “Distance” parameter (Fig 2b), the same trends in the data are also observed in the alteration of color preference with the same change in direction but to a different extent. The alcohol treatments (both the acute and chronic) increased thigmotaxis by minimizing the distance between the zebrafish and the CPP wall in red-related color combinations (Fig 2c). Concentration-dependent effects were found in locomotor activity-related parameters in each color combination (Fig 2d–2g), which were affected by the acute and chronic alcohol treatments. Generally, the locomotive parameters in the acute alcohol treatment exhibited a “V” or “inverted V” pattern with the acute 0.5% or 1.0% treatments as the extreme value. Specifically, the analysis of velocity showed that acute 0.5% increased the velocity value at first and then 1.0% and 1.5% decreased the velocity compared with the control while the chronic 1.0% treatment significantly decreased the velocity value compared with the acute treatment and the control. Similar numerical changes in the behavioral parameters were also found in “Turning angle” (Fig 2e), “Angle velocity” (Fig 2f), “Freezing time ratio” (Fig 2g), “Swimming time ratio” (Fig 2h), and “Rapid move time ratio” (Fig 2i), indicating the different effects of the acute vs. chronic alcohol treatments on locomotor activity.

Judging from the variation in color preference order, acute alcohol exposure enhanced the preference of zebrafish towards red in the color-enhanced CPP test, thus changing the color associated with the learning and memory patterns of zebrafish. Among all of the treatment groups, acute 1.0% changed learning and memory most significantly. For the other parameters that reflect locomotion and coordination, the dose effect patterns among all of the experiment groups were similar with 1.0% exerting the most obvious effect. Importantly, acute 1.0% was a significant turning point in the trends in the variation of most of the advanced behavioral parameters, thus generating an ‘inverted V’ dose-dependent pattern in all of the experiment groups.

### Physiological mechanisms of behavioral changes in associated learning and memory based on color preference and Parkinson-like swimming patterns

**Fig 3:**
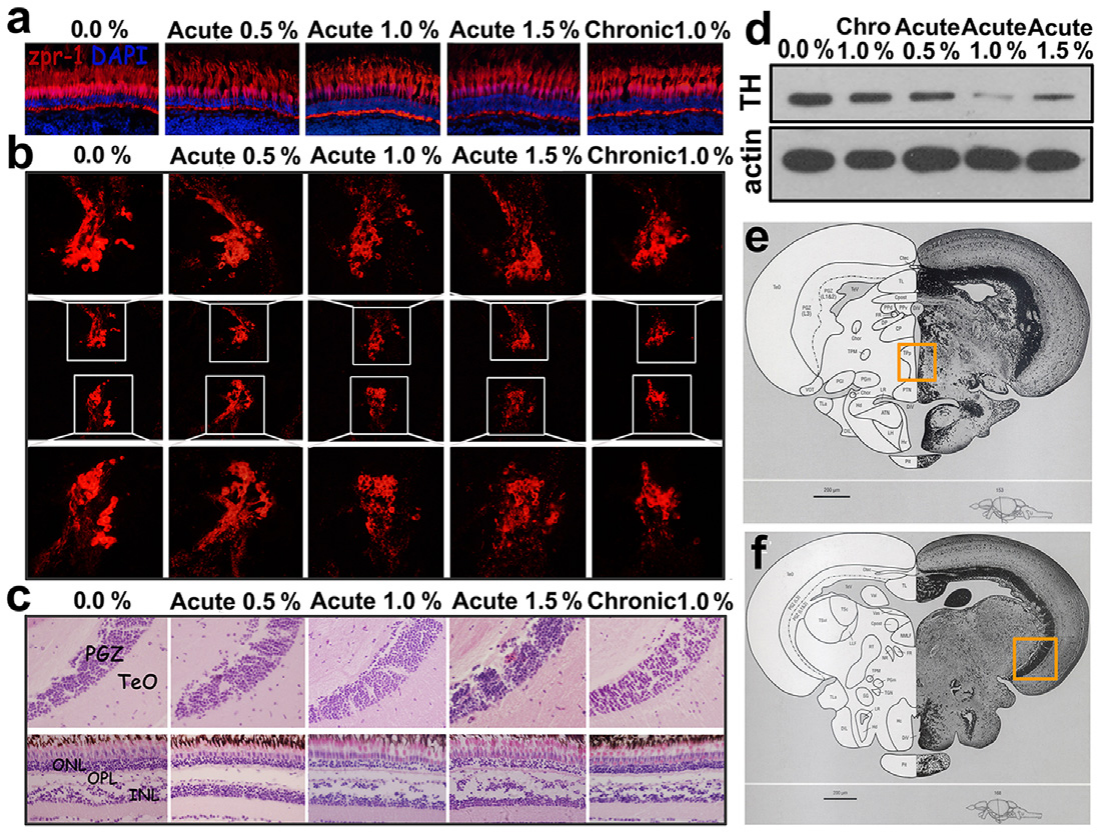
The effects of acute and chronic alcohol exposure on the retina and brain of the zebrafish. **(a)** Cone photoreceptor cells labelled by the Zpr-1 antibody in wild-type zebrafish and zebrafish groups treated with alcohol. **(b)** Tyrosine hydroxylase (TH) expression in the posterior tuberculum (PT) detected by immunohistochemistry and western blotting. **(c)** The histopathology of the brain and retina in wild-type zebrafish and in the groups treated with alcohol. **(d)** TH expression in the brain detected by western blotting. **(e)** The boxed area in the section from the adult zebrafish brain highlighting the brain region shown in B. **(f)** The boxed area in the section from the adult zebrafish brain highlighting the brain region shown in C. Cropped blots are used in the figure, and full-length blots are presented in **Fig J** in S1 File. The images in E and F are reproduced from Wullimann, M., Neuroanatomy of the Zebrafish Brain: A topological atlas, Birkhauser press, Basel (1996)36, Chapter 5, Fig 153 and Chapter 5 Fig 168, respectively, with permission from Springer Science and Business Media.

Because the alcohol treatments disturbed color preference in the zebrafish, we examined the expression of Zpr-1 in the cone cells, which are the photoreceptors responsible for color vision in the zebrafish retina [51]. The monoclonal antibody (mAb) Zpr-1 is specifically associated with the antigen in the plasma membrane of double cone cells [52]. As shown in Fig 3a, no obvious difference in morphology was found between the wild-type group and the alcohol treated groups, but the level of Zpr-1 expression in the alcohol-treated groups was less than that in the wild-type group. Additionally, the number of double cone cells was less than in the wild-type zebrafish.

Decreased locomotive activity and abnormal motion patterns were also observed in the alcohol-treated zebrafish; these are behaviors characteristic of Parkinson’s disease [53], which results from the progressive loss of dopaminergic neuron (DA) cells in the substantia nigra (Fig 3b). To study whether the observed decrease in locomotive activity was related to a loss of DA cells, we performed immunohistochemistry and western blotting to examine the expression of tyrosine hydroxylase (TH). The results showed that alcohol decreased the expression of TH in the posterior tuberculum (PT) in the brain of zebrafish [54], and the levels of TH in the groups treated with acute 1.0% were much lower than in the wild-type group (Fig 3d). In addition, HE staining was performed to examine the histopathology of the brain and retina in zebrafish following exposure to alcohol (Fig 3c). No apparent histologic changes were observed in the tissues of the brain or retina in the alcohol-treated groups and the wild-type group.

## Discussion

The deviation of the color preference parameters from the normal values and the changes in the color preference patterns of the zebrafish treated with alcohol demonstrated that alcohol could disturb the natural mode of visual-based learning and memory in adult zebrafish. Similar to the human retina, the zebrafish retina is composed of highly organized neural tissue, and the retinal photoreceptors are the primary sensory cells responsible for detecting light. The zebrafish retina also contains both rod and cone photoreceptor cells; rod photoreceptor cells mediate dim-light and night vision, whereas cone photoreceptor cells are responsible for mediating bright light and color vision in the daytime [51]. In this study, mAb and Zpr-1 were used to label the cone photoreceptor cells to determine whether alcohol exposure could disturb the natural mode of color preference. No obvious difference in morphology was found between the wild-type group and the alcohol treated groups, but the expression level of Zpr-1 in the alcohol-treated groups was less than that in the wild-type group. The number of double cone cells in the alcohol-treated groups was less than in the wild-type zebrafish. The histopathological features of the retina were also examined by HE staining, and no apparent difference was found between the wild-type group and the treated groups. Together, these data suggest that the disturbed color preference exhibited by the four alcohol-treated zebrafish groups was likely due to a reduction in cone photoreceptors, which may have been caused by the alcohol treatment.

Both acute and chronic alcohol treatments result in decreased locomotive activity in zebrafish, which is evidenced by decreased locomotive parameter values, such as distance, velocity and freezing time ratio. Decreased locomotive activity has been reported to be the primary representative behavioral alteration of Parkinson’s disease exhibited by the zebrafish model [53], and the progressive loss of DA neurons in the substantia nigra is characteristic of Parkinson’s disease [55]. In the alcohol-treated zebrafish, the DA neurons were reduced in the posterior tuberculum (PT), the counterpart of the substantia nigra in amniotes. Together, the data suggest that alcohol could induce the characteristics of Parkinson’s disease in zebrafish, and the decreased locomotive activity observed in the treated zebrafish was caused, at least in part, by the loss of DA cells in the posterior tuberculum. The four groups of zebrafish treated with alcohol of different concentrations exhibited DA cell loss to different extents, which may be the reason for the variations in the degree to which their locomotive activity was decreased. It has been reported that alcohol exposure can reduce dopamine release in the nucleus accumbens core of adult rats [52], and dopamine innervation density was found to be lower in the NAc of rats that preferred alcohol compared with those that did not [54, 56]. This, combined with our results in this study, suggests that there is a complicated interaction between the dopaminergic system and alcohol either through alcohol neurotoxicity or alcohol consumption.

In conclusion, considering all of the parameters, including color preference and locomotive activity as well as the spatiotemporal swimming patterns of zebrafish, the neurotoxic order of the alcohol treatments was acute 1.0%>acute 1.5%>acute 0.5%> chronic 1.0%>0.0%. Within the acute alcohol treatment, the 1.0% treatment had the greatest anxiety-relieving effect, but under this euphoric emotional state, visual associative learning and memory and locomotive patterns change dramatically in contrast to the acute 0.5% and 1.5% and chronic 1.0% treatments. However, compared with the acute treatment, chronic 1.0% alcohol exposure increases anxiety, but it has a moderate effect on learning and memory and locomotive activity. Furthermore, the acute treatment has greater potential to cause Parkinson-like behaviors in zebrafish, so the biological mechanism must be further researched in depth.

## Supporting Information

S1 File Experimental strategy. Comparison of Manually Labeled Trajectories and Automated Trajectories (**Fig A**). Histogram of tracking error per frame (**Fig B**). Thigmotaxis Definition (**Fig C**). Comparison of automated and manual trajectory (**Fig D**). Flowchart illustrating the experimental strategy of this research (**Fig E**). Reflection spectral characterization of the four colour (blue, green, yellow, red) used in associated learning and memory test based on colour preference (**Fig F**). Full length blots(**Fig J**).
(DOC)
