# Supplementary material for: The Difference between Anxiolytic and Anxiogenic Effects Induced by Acute and Chronic Alcohol Exposure and Changes in Associative Learning and Memory Based on Color Preference and the Cause of Parkinson-Like Behaviors in Zebrafish
Source: PLoS One. 2015 Nov 11;10(11):e0141134. doi: 10.1371/journal.pone.0141134 (PMC4641683; doi:10.1371/journal.pone.0141134)
Supplement: S1 File — Comparison of Manually Labeled Trajectories and Automated Trajectories (Fig A). Histogram of tracking error per frame (Fig B). Thigmotaxis Definition (Fig C). Comparison of automated and manual trajectory (Fig D). Flowchart illustrating the experimental strategy of this research (Fig E). Reflection spectral characterization of the four colour (blue, green, yellow, red) used in associated learning and memory test based on colour preference (Fig F). Full length blots(Fig J). (DOC) [file pone.0141134.s001.doc]

**Supplementary materials**

**The difference between** **anxiolytic and anxiogenic effects induced by acute and chronic alcohol exposure and changes in associative learning and memory based on color preference and the cause of Parkinson-like** **behaviors in zebrafish**

**Xiang Li^1^**^¶^**, Xu Li^3^**^¶^**, Yi-Xiang Li^1^**^¶^**, Yuan Zhang^1^, Di Chen^2^, Ming-Zhu Sun^2^, Xin Zhao^2*^, Dong-Yan Chen^3*^, Xi-Zeng Feng^1*^**

^1^State Key Laboratory of Medicinal Chemical Biology, Key Laboratory of Bioactive Materials, Ministry of Education, College of Life Science, Nankai University, Tianjin 300071, China,

^2^The Institute of Robotics and Automatic Information Systems, Nankai University, Tianjin 300071, China,

^3^The Key Laboratory of Animal Models and Degenerative Diseases, Department of Physiology, School of Medicine, Nankai University, Tianjin, 300071, China.

^¶^ These authors contributed equally to this work.

^*^Correspondence should be addressed to X.Z.(zhaoxin@nankai.edu.cn), D.Y.C. (chendy@nankai.edu.cn), X.Z.F. (xzfeng@nankai.edu.cn)

**Contents**

**Supplementary Methods**

**Supplementary Figure**

**Supplementary Reference**

**SUPPLEMENTARY METHODS**

1. **Self-designed video-tracking Software introduction for light and dark box**

We have designed a Zebrafish Tracking software based on self-organizing background subtraction and blob tracking algorithm. Kalman Filter is utilized for location prediction and matching in blob tracking algorithm. The software is born for high-throughput, precise and robust single adult Zebrafish tracking which places firm basis for further research in neuro-ethological experiment. It has been tested in color performance experiment and works well for white-black experiment. The automated tracking result is quantitative compared with manually labeled trajectory according to Supplementary Figure S1. Specifically, the speed of our software is 5.6869 FPS using 400×300 size video, the tracking error per frame is 0.3567 centimeters and more than 90% tracking error is lower than 0.5 centimeters according to. The test is carried on using experiment video which reflects the natural experiment environment including disturbance. All in all, the quantitative experiments have proved that our tracking software is capable of high-throughput and complicated research.

Moreover, based on high-throughput and precise tracking, our self-designed zebrafish tracking software mined more sophisticated behavioral features including speed, total distance, turn angle, turn angle velocity, counting the times of crossing white and black, duration to the first time to white/black, duration and frequency of immobile swim, stay time in white part and [thigmotaxis](app:ds:thigmotaxis). We regard the instantaneous speed < 1 cm/s as immobile swim, 1cm/s < instantaneous speed < 20 cm/s as normal swim and instantaneous speed > 20 cm/s as rapid swim. As to [thigmotaxis](app:ds:thigmotaxis), we divide our container into 4 part according to Figure S2 We count how long times zebrafish is less than 2 cm to nearest wall as [thigmotaxis](app:ds:thigmotaxis)（Fig.S3）. These sophisticated behavioral features provide effective tools to evaluate neuro-behaviors of zebrafish.

**2. Self-designed video-tracking Software introduction for color-enriched conditional place preference**

We utilized self-designed Zebrafish tracking software. Aiming at precise tracking of single adult Zebrafish, the self-organizing background subtraction and blob tracking algorithmwhich is effective for single target tracking are integrated and implemented. Based on precise moving object (Zebrafish) detection, the blob tracking algorithms use Kalman Filter for Zebrafish location prediction and matching. Our algorithms perform precise, high-throughput and robust tracking which is capable of further behavioral research. To quantify the performance of software, our results are compared with manually labeled trajectories (at the same time stamp concerned about the manually labeled trajectory is sparse) according to **Supplementary Figure S1.** And the tracking error per frame is 0.3567 centimeters and over 90% tracking error is below 0.5 centimeters, according to **Supplementary Figure S2,** in comparison with manual trajectories according to 40-minute test video which covers the possible disturbance in our experiment environment. Moreover, the speed of our software is 5.6869 FPS. Hence, the accuracy and speed of self-designed Zebrafish tracking software is qualified for high-throughput and complex behavioral research.

Besides its accuracy and speed, automated Zebrafish tracking software enjoys indisputable advantages over manual trajectories of trajectory density which stands for more abundant behavior information. Furthermore, based on such precise and dense trajectory, very complex behavior features containing in swimming distance, velocity, stay time, freezing, swimming, rapid movement detection, [thigmotaxis](app:ds:thigmotaxis), turn angle and turn angle velocity are quantified and brought into research. Such complex features are capable of quantifying the neurobehavioral parameters such as locomotive activities and color preference for further analysis.

Among all the features, distance, stay time and velocity are calculated directly from trajectories. The locomotive status, based on [instantaneous](app:ds:instantaneous)[velocity](app:ds:velocity), is classified into three categories, freezing for velocity less than 1 cm/s, swimming for velocity between 1 cm/s and 10 cm/s and rapid movement for velocity more than 10cm/s. The [thigmotaxis](app:ds:thigmotaxis)is defined as the distance of detected Zebrafish to nearest wall of container during 3minuts test. The turning angle and turn angle velocity are calculated through intersection angle between continuous two moving vectors

$\begin{matrix} \overrightarrow{V_{t}}=(x_{t}-x_{t-1},y_{t}-y_{t-1}) \\ \overrightarrow{V_{t+1}}=(x_{t+1}-x_{t},y_{t+1}-y_{t}) \\ \cos(A_{t})=\frac{\overrightarrow{V_{t}}\bullet\overrightarrow{V_{t+1}}}{\left|\overrightarrow{V_{t}}\right|\bullet\left|\overrightarrow{V_{t+1}}\right|} \end{matrix}$ .

Amongst, $(x_{t},y_{t})$ is coordinate of Zebrafish at time $t$ and $A_{t}$ is turning angle. The [instantaneous](app:ds:instantaneous) features are used for visualization and they are averaged for statistically analyzed. On the other hand, the comparison between average features in left and right in two colored compartments in CPP serves as direct way to quantify the Alcohols influence on Zebrafish’s color preference and locomotive activities.

**SUPPLEMENTARY FIGURE**


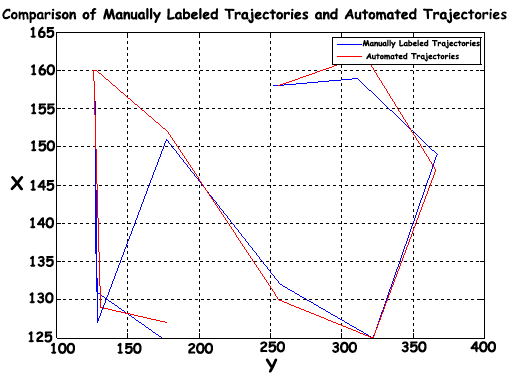


**Supplementary Figure S1:** Comparison of Manually Labeled Trajectories and Automated Trajectories


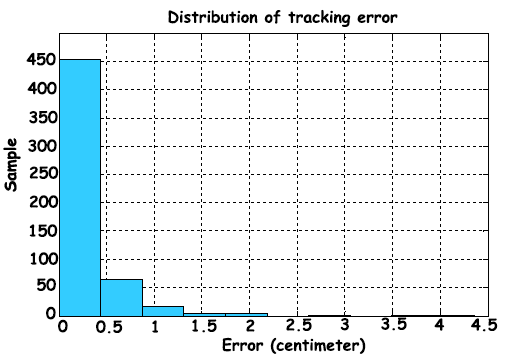


**Supplementary Figure S2:** Histogram of tracking error per frame


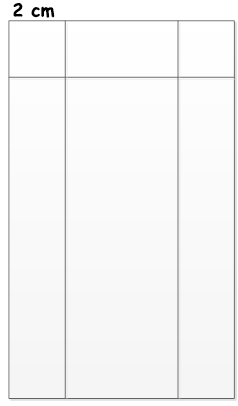


**Supplementary Figure S3:** Thigmotaxis Definition


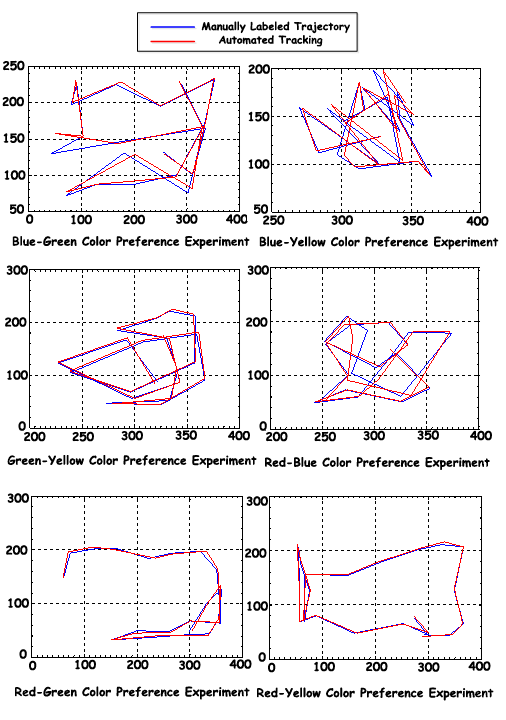


**Supplementary Figure S4:** Comparison of automated and manual trajectory


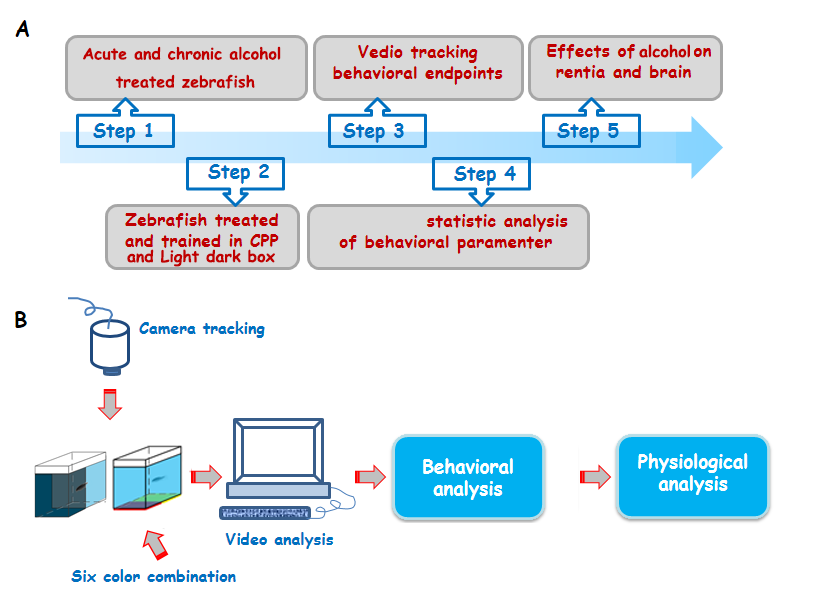


**Supplementary Figure S5:** Flowchart illustrating the experimental strategy of this research. The illustration (A) includes Alcohols treated zebrafish in standard tank water (Step 1). Zebrafish were trained in CPP tank and dosed in habitual tanks for seven days before tested (Step 2). During the process of test, 9 behavioral endpoints as well as the swimming path of zebrafish were recorded by video (Step 3). Subsequently, statistical analysis were conducted across all the behavioral parameters and experimental treatments in order to discover potential size and concentration effect of Alcohols over neural behavior of zebrafish (Step 4).Physiological analysis were performed to 9 detect the effect of Alcohols on the retina and brain (Step 5). (B) Standard procedure of light and dark preference test and color preference test. For light and dark preference test, each zebrafish were recorded by camera for 5min.For colour preference test, trained wild-type zebrafish were tested in CPP tank with six color combination in the bottom, and for each color combination zebrafish were tested in 3 minutes, which were recorded by video. Animal behavior was automatically observed and one camera recorded videos for automated analysis in self-designed software for zebrafish tracking. Raw data obtained from tracking software were analyzed via statistics.


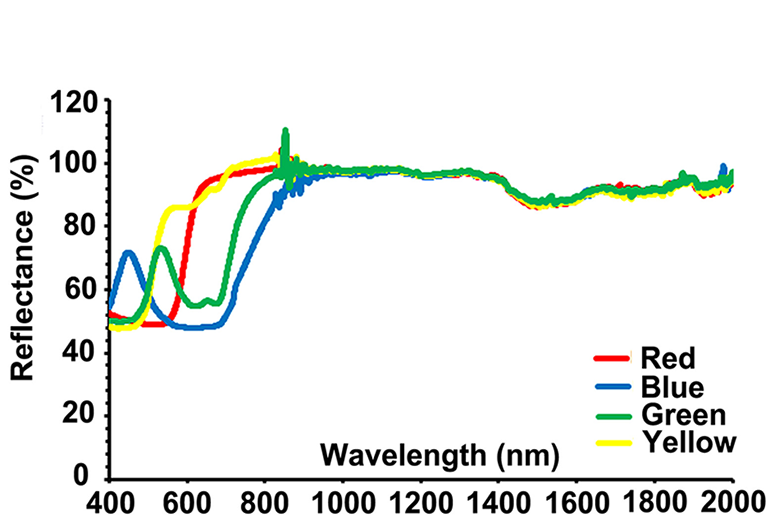


**Supplementary Figure S6:** Reflection spectral characterization of the four colour (blue, green, yellow, red) used in associated learning and memory test based on colour preference.
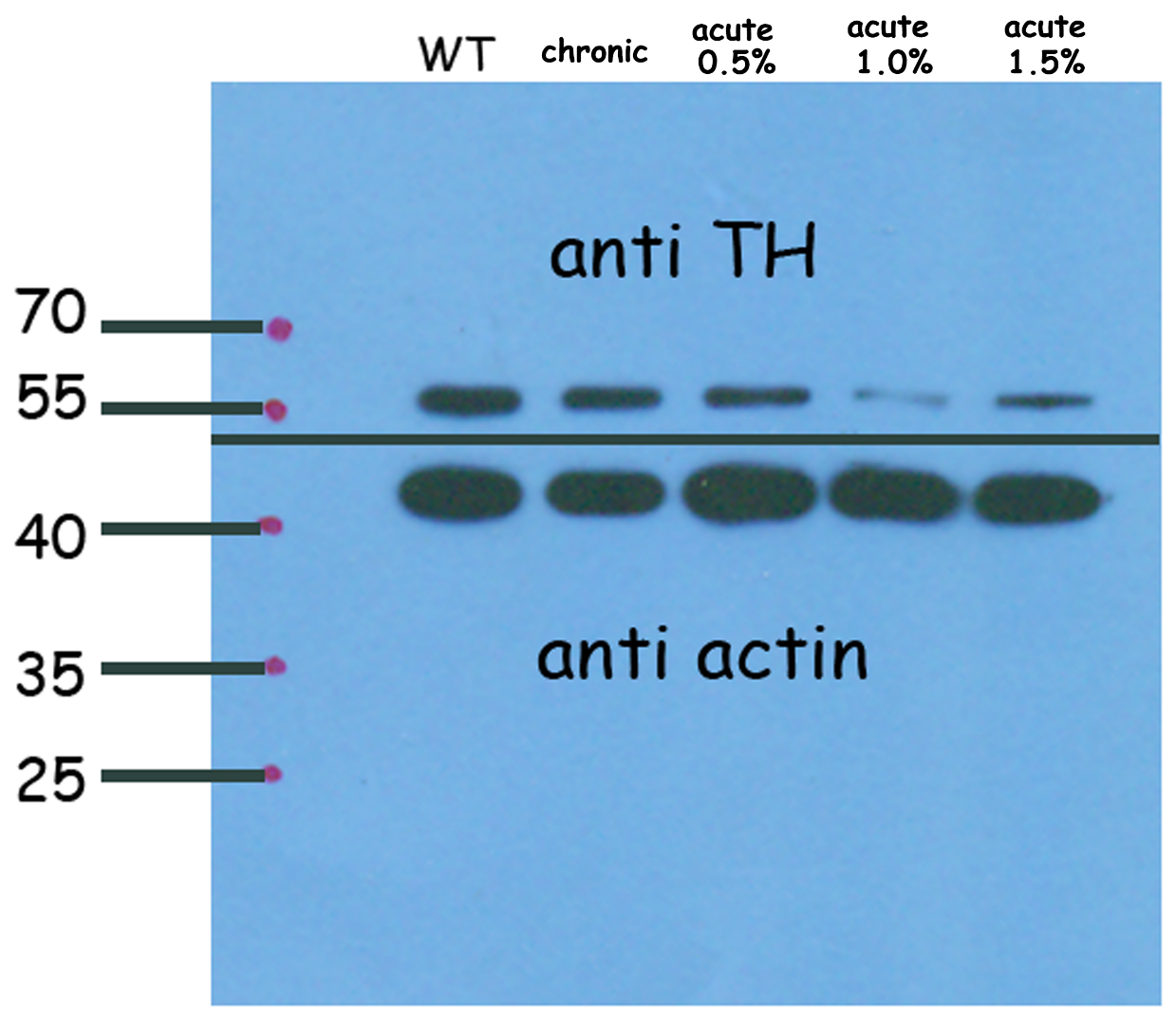


**Supplementary Figure S7: Full-length blots.**

**SUPPLEMENTARY REFFERNCE**

1. Maddalena, L. and A. Petrosino, *A self-organizing approach to background subtraction for visual surveillance applications.* Image Processing, IEEE Transactions on, 2008. **17**(7): p. 1168-1177.

2. Maddalena, L. and A. Petrosino. *The SOBS algorithm: what are the limits?* in *Computer Vision and Pattern Recognition Workshops (CVPRW), 2012 IEEE Computer Society Conference on*. 2012. IEEE.

3. Gavrila, D.M., *The visual analysis of human movement: A survey.* Computer vision and image understanding, 1999. **73**(1): p. 82-98.

4. Sobottka, K. and I. Pitas, *A novel method for automatic face segmentation, facial feature extraction and tracking.* Signal processing: Image communication, 1998. **12**(3): p. 263-281.
